# Supplementary material for: “Corp-Osa-Mente”, a Combined Psychosocial–Neuropsychological Intervention for Adolescents and Young Adults with Fragile X Syndrome: An Explorative Study
Source: Brain Sci. 2023 Feb 7;13(2):277. doi: 10.3390/brainsci13020277 (PMC9953950; doi:10.3390/brainsci13020277)
Supplement: Supplementary file 1 [file brainsci-13-00277-s001.zip › brainsci-2089046-supplementary.pdf]

THE SURVEY

Please respond to the following questions by placing a check mark (√) in the answer box that corresponds to your response and/or fill in the blank where indicated. Answers are anonymous.

The compiler is:

o Female participant to the project

o Male participant to the project

o Father

o Mother

o other family member. Please specify:

o A professional working with the participant (e.g., Educator). Please specify:

Is the participant for whom you are answering/ are you carrying out any other treatments? If yes, which ones?

o Pharmacological

o Psycho-educational with non-psychologist educator

o Psycho-educational with another psychologist

o Other. Please specify:

Are you, on average, satisfied with the project?

o Yes

o No

Areas of improvement. You can choose more than one answer.

o Autonomy in the home environment (e.g. preparing meals alone)

o Autonomy in the social environment (e.g. going out with friends)

o Autonomies in the work environment (e.g. organising one's own work)

o Autonomies at school (e.g. greater management of study materials)

o Receptive language skills (e.g. greater ability to understand others)

o Expressive language skills (e.g. improved speech, expanded vocabulary)

o Reading-writing skills

o Attention, memory and executive functions (e.g. increased attentional skills, increased ability to remember and plan commitments)

o Behaviour management (e.g. anger outbursts)

o Emotion management (e.g. anxiety, fear, sadness)

o Ability to communicate one's emotional states

o Other. Please specify:

Areas of null-effects. You can choose more than one answer.

o Autonomy in the home environment (e.g. preparing meals alone)

o Autonomy in the social environment (e.g. going out with friends)

o Autonomies in the work environment (e.g. organising one's own work)

o Autonomies at school (e.g. greater management of study materials)

o Receptive language skills (e.g. greater ability to understand others)

o Expressive language skills (e.g. improved speech, expanded vocabulary)

o Reading-writing skills

o Attention, memory and executive functions (e.g. increased attentional skills, increased ability to remember and plan commitments)

o Behaviour management (e.g. anger outbursts)

o Emotion management (e.g. anxiety, fear, sadness)

o Ability to communicate one's emotional states

o Other. Please specify:

Which aspect(s) do you like the most about the intervention? You can choose more than one answer.

o On-person meetings

o Online meetings

o The possibility of having a WhatsApp group to interact in

o The relationship with the psychologist

o Homework between meetings

o Parenting meetings

o Other. Please specify:

Which aspect(s) do you like the least about the intervention? You can choose more than one answer.

o On-person meetings

o Online meetings

o The possibility of having a WhatsApp group to interact in

o The relationship with the psychologist

o Homework between meetings

o Parenting meetings

o Other. Please specify:

Which techniques do you find the most effective?

O Psychoeducation

o Mindfulness

o Cognitive techniques (ABC method)

o Neuropsychological techniques (cognitive training)

o Behavioral techniques

o Counselling

o Time management

o Leisure activities

o Occupational therapy

o Sexual education

Family members only. Since when the participant is in the project (you can choose more than one answer):

o I can interact more easily with her/him

o I can understand his/ her emotions more easily

o I feel less "stressed

o All of the above

o None of the above

o Other personal ideas about the impact the project is having on you as a parent:_____________

Educators only. Since the project started (you can choose more than one answer):

o I know that I can interact with an expert in case of difficulties with the individual with FXS

o I can more easily understand the emotions of the individual with FXS

o I have a clearer idea of FXS

o All of the above

o None of the above

o Other. Please specify:____

Some suggestions:

____
____
____
